# Machine learning-based mortality prediction model for heat-related illness

**DOI:** 10.1038/s41598-021-88581-1

**Published:** 2021-05-04

**Authors:** Yohei Hirano, Yutaka Kondo, Toru Hifumi, Shoji Yokobori, Jun Kanda, Junya Shimazaki, Kei Hayashida, Takashi Moriya, Masaharu Yagi, Shuhei Takauji, Junko Yamaguchi, Yohei Okada, Yuichi Okano, Hitoshi Kaneko, Tatsuho Kobayashi, Motoki Fujita, Hiroyuki Yokota, Ken Okamoto, Hiroshi Tanaka, Arino Yaguchi

**Affiliations:** 1 Department of Emergency and Critical Care Medicine, Juntendo University Urayasu Hospital, Tomioka, 2-1-1, Urayasu, Chiba 279-0021 Japan; 2 Department of Emergency and Critical Care Medicine, St. Luke’s International Hospital, Tokyo, Japan; 3 Department of Emergency and Critical Care Medicine, Nippon Medical School, Tokyo, Japan; 4 Department of Emergency Medicine, Teikyo University Hospital, Tokyo, Japan; 5 Department of Traumatology and Acute Critical Medicine, Osaka University Graduate School, Suita, Osaka, Japan; 6 Department of Emergency Medicine, North Shore University Hospital, Northwell Health System, Manhasset, NY USA; 7 Department of Emergency and Critical Care Medicine, Jichi Medical University Saitama Medical Center, Saitama, Japan; 8 Department of Emergency, Disaster and Critical Care Medicine, Showa University School of Medicine, Tokyo, Japan; 9 Department of Emergency Medicine, Asahikawa Medical University Hospital, Asahikawa, Hokkaido, Japan; 10 Department of Acute Medicine, Nihon University School of Medicine, Tokyo, Japan; 11 Department of Primary Care and Emergency Medicine, Graduate School of Medicine, Kyoto University, Kyoto, Japan; 12 Department of Emergency Medicine, Japanese Red Cross Kumamoto Hospital, Kumamoto, Japan; 13 Emergency and Critical Care Center, Tokyo Metropolitan Tama Medical Center, Tokyo, Japan; 14 Department of Emergency and Critical Care Medicine, Aizu Chuo Hospital, Aizuwakamatsu, Fukushima, Japan; 15 Advanced Medical Emergency and Critical Care Center, Yamaguchi University Hospital, Ube, Yamaguchi, Japan; 16 Department of Critical Care and Emergency Medicine, Tokyo Women’s Medical University, Tokyo, Japan

**Keywords:** Health care, Medical research

## Abstract

In this study, we aimed to develop and validate a machine learning-based mortality prediction model for hospitalized heat-related illness patients. After 2393 hospitalized patients were extracted from a multicentered heat-related illness registry in Japan, subjects were divided into the training set for development (n = 1516, data from 2014, 2017–2019) and the test set (n = 877, data from 2020) for validation. Twenty-four variables including characteristics of patients, vital signs, and laboratory test data at hospital arrival were trained as predictor features for machine learning. The outcome was death during hospital stay. In validation, the developed machine learning models (logistic regression, support vector machine, random forest, XGBoost) demonstrated favorable performance for outcome prediction with significantly increased values of the area under the precision-recall curve (AUPR) of 0.415 [95% confidence interval (CI) 0.336–0.494], 0.395 [CI 0.318–0.472], 0.426 [CI 0.346–0.506], and 0.528 [CI 0.442–0.614], respectively, compared to that of the conventional acute physiology and chronic health evaluation (APACHE)-II score of 0.287 [CI 0.222–0.351] as a reference standard. The area under the receiver operating characteristic curve (AUROC) values were also high over 0.92 in all models, although there were no statistical differences compared to APACHE-II. This is the first demonstration of the potential of machine learning-based mortality prediction models for heat-related illnesses.

## Introduction

Rising global temperatures owing to excessive carbon dioxide emissions or heat island effect caused by urbanization have been endangering human health worldwide^1,2^. Increase in the aging population, which is vulnerable to the health effects of heat, has also enhanced the occurrence of heat-related diseases^3^. Although a large number of studies over the decades has revealed the epidemiology, risk factors, and preventative management of such diseases, reducing the occurrence of heat-related illness is challenging because it requires solutions by society as a whole, such as installation of air conditioners for the elderly or low-income citizens. In fact, numerous instances of hospitalization and eventual death of patients suffering from heat-related illness continue to be recorded. During 2014–2018, death due to heat-related illnesses in the United States was reported to be an average of 702 per year^4^. In this background, medical practitioners are continuously challenged to generate high quality of care for heat-related illness.

The most important treatment for heat-related illness is rapid and effective cooling. There are various cooling strategies such as cold-water immersion, administration of cold fluids, application of ice packs or wet gauze sheets, fanning, and cooling suits^2,5^. In addition, more invasive methods are selected for critical patients, such as an intravascular cooling device or extracorporeal circulatory support system^6,7^. Occasionally, artificial ventilation, hemodialysis, or liver transplantation might be necessary for organ support^8,9^. However, it is difficult for clinicians to optimize therapeutic intervention according to individual patient conditions. The availability of clinical prognostic tools could be helpful in deciding these treatment options. Furthermore, the prognostic model could be used retrospectively to assess the quality of care for heat-related illness.

In recent years, prognostic tools using machine learning have been widely developed and applied in medicine, as they often outperform conventional prediction methods^10^. In contrast, a machine learning-based mortality prediction model for heat-related illness has not been developed previously. In this study, we aimed to develop and validate machine learning-based mortality prediction models for use in hospitalized patients with heat-related illnesses.

## Methods

### Data sources and ethical approval

The data for this retrospective cohort study were obtained from the “Heatstroke study” database in Japan. A heatstroke study was undertaken by the Japanese Association for Acute Medicine (JAAM) to clarify the epidemiology of heat-related illness in Japan. The data were manually recorded by a staff member or medical doctor at each participating hospital using specific record sheets. From 2014, patients with heat-related illness who were admitted to the hospitals were included in the heatstroke study, except for the period 2015–2016, in which the heatstroke study was not conducted. Diagnosis of heat-related illness was based on the judgement of the clinician in each participating hospital. Thus, data from the heatstroke studies in 2014 and 2017–2020, from 109 to 142 participating hospitals, were extracted for our study. The heatstroke study has been described elsewhere^11,12^.

The heatstroke study protocol was approved by the ethics committee of Showa University Hospital. Patient information was de-identified before being provided for use in this study. The requirement for patient informed consent was waived, as this was an observational study using anonymous data. The current study was conducted in accordance with the Declaration of Helsinki.

### Study population

**Figure 1:**
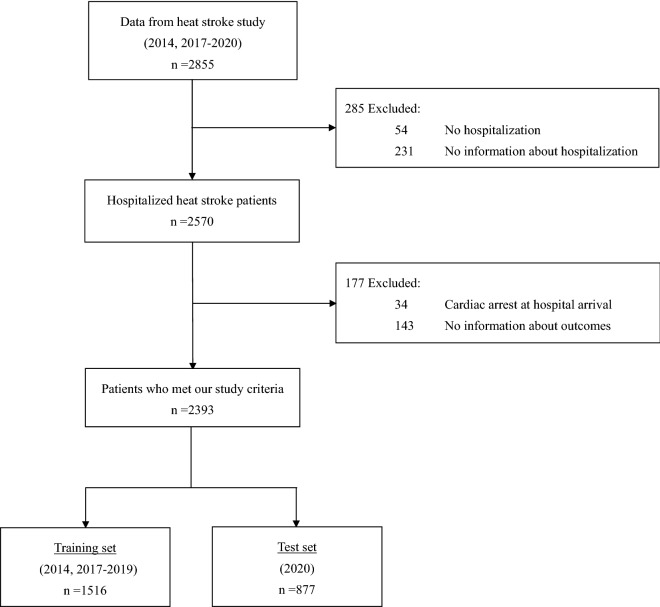
Flow diagram of patient inclusion procedure.

Overall, 2855 patients with heat-related illness were identified from the heatstroke study data in 2014 and 2017–2020. Of these, 285 patients were excluded because they were not hospitalized or no information was available regarding their hospitalization. Further, cases with cardiac arrest at hospital arrival and incomplete data regarding survival outcome were excluded. In total, the data of 2393 patients hospitalized with heat-related illness met the inclusion criteria. Finally, the subjects were classified into two groups: training set (n = 1516, data from 2014, 2017–2019) and test set (n = 877, data from 2020) (Fig. 1).

### Outcome and variable selection

In this study, the outcome was set as death during hospital stay. From the heatstroke study database, 24 variables with missing values below 25% of all samples were extracted as predictor features for the outcome. These variables were age, sex, location at the onset (indoor or outdoor), vital signs (systolic blood pressure, diastolic blood pressure, heart rate, respiratory rate, and body temperature), total Glasgow coma scale (GCS), peripheral oxygen saturation (SpO_2_), and laboratory data [pH, base excess, hematocrit, platelet count, blood urea nitrogen (BUN), creatinine, total bilirubin, aspartate aminotransferase (AST), alanine aminotransferase (ALT), creatine kinase, sodium, potassium, glucose, and prothrombin time/international normalized ratio (PT-INR)] at patients’ hospital arrival. Missing data were imputed from the median of each variable.

### Development of machine learning models

Four kinds of machine learning models including logistic regression, support vector machine, random forest, and XGBoost were trained by using variables selected for mortality prediction in the training set. First, feature scaling to normalize the range of independent variables was accomplished. In the process of training, tenfold stratified cross-validation was used to avoid overfitting of the model. In short, the training data were partitioned into 10 stratified subsets. Subsequently, 9 subsets (90% of training data) were used to train the model, and the remaining subset (10% of training data) was used for the validation. These training and validation processes were repeated 10 times with each of the subsets used once as a validation dataset, allowing us to obtain 10 estimates of predictive accuracy, which were averaged to obtain a single estimate. Because our data were imbalanced for the outcome, we used cost-sensitive learning. In addition, optimization of hyperparameters (values that control the machine learning process) was performed for each model (Supplementary Table 1).

To assess the feature importances for the model development, Gini importances were computed as the normalized total reduction of the criterion brought by the feature for random forest and XGBoost models. For the logistic regression model, absolute values of standardized beta coefficients were described.

### Validation of developed machine learning models

The performance of the developed machine learning models was validated using the test data; this process was independent of the algorithm training process. We compared these models with the conventional acute physiology and chronic health evaluation (APACHE)-II score as the reference standard for prediction of the outcome. The area under the receiver operating characteristic curve (AUROC), the area under the precision-recall curve (AUPR), sensitivity, specificity, positive predictive value (PPV), negative predictive value (NPV), and accuracy were measured as the performance indicators. To observe the correlation between predicted and observed probabilities of mortality during hospital stay, we created calibration plots in the test set.

### Libraries for data analyses and machine learning

To present the patient data, the mean with standard deviation (SD) or median with interquartile range (IQR) was used for the numerical variables. For categorical variables, counts with percentages were reported. For comparison analysis between two samples, the t-test and Mann–Whitney U test were used for the means and medians of samples, respectively. The frequencies were compared using the chi-square test. The two-sided significance level for all tests was set at 5% (p < 0.05). Patient characteristics were analyzed using the SciPy (version 1.5.2) with Python (version 3.7.4 in Anaconda 2019.10). Development of machine learning models was employed by Scikit-learn (version 0.21.3) with Python.

## Results

### Characteristics of study subjects

The baseline characteristics of the included patients are shown in Table 1. The mean age of all included patients was 65 ± 22 years, and 70.4% of the patients were men. Outdoor heat-related illness accounted for 54.9% of all patients. The mortality rate during hospital stay was only 5.2%, indicating that the analyzed dataset was highly imbalanced for the outcome. In comparison between training and test dataset, there were significant differences for age, location at the onset, body temperature, SpO_2_, pH, BUN, creatinine, total bilirubin, creatine kinase, and sodium. However, most of these differences appear to be clinically irrelevant.

**Table 1:** Baseline characteristics of the study population.

Variables	All (n = 2393)	Missing	Training data (n = 1516)	Test data (n = 877)	P value
Age (years)	65 ± 22	3	64 ± 22	68 ± 21	< 0.01
Gender (male)	1678 (70.4%)	9	1060 (70.0%)	618 (71.1%)	0.78
Location at the onset (outdoor)	1290 (54.9%)	45	853 (57.8%)	437 (50.1%)	< 0.01
**Vital signs at hospital arrival**					
Systolic blood pressure (mmHg)	126 ± 31	34	126 ± 32	125 ± 31	0.24
Diastolic blood pressure (mmHg)	75 ± 21	50	75 ± 21	75 ± 21	0.48
Heart rate (beats/min)	105 ± 28	21	105 ± 29	105 ± 28	0.83
Respiratory rate (/min)	25 ± 9	200	25 ± 9	24 ± 9	0.28
Body temperature (℃)	38.1 ± 1.6	214	38.1 ± 1.8	38.2 ± 1.6	0.03
Total GCS	14 (10–15)	44	14 (10–15)	14 (10–15)	0.99
SpO_2_ (%)	97 ± 4	70	97 ± 5	97 ± 3	0.02
**Laboratory data**					
pH	7.42 ± 0.1	415	7.42 ± 0.1	7.41 ± 0.2	< 0.01
Base excess (mmol/L)	− 2.2 ± 4.7	464	− 2.3 ± 5.2	− 2.3 ± 5.3	0.32
Hematocrit (%)	40.8 ± 7.3	67	40.8 ± 7.2	40.9 ± 7.8	0.49
Platelet count [unit ten thousand (/μL)]	22.7 ± 14.0	68	23.0 ± 16.4	22.3 ± 9.3	0.87
BUN (mg/dL)	29.7 ± 20.8	43	28.6 ± 19.3	31.9 ± 23.3	< 0.01
Creatinine (mg/dL)	1.8 ± 1.6	30	1.8 ± 1.6	1.9 ± 1.6	0.02
Total bilirubin (mg/dL)	1.2 ± 0.9	86	1.1 ± 0.9	1.2 ± 1.0	< 0.01
AST (IU/L)	72 ± 177	30	73 ± 194	71 ± 147	0.11
ALT (IU/L)	44 ± 104	29	45 ± 111	42 ± 93	0.51
Creatine kinase (IU/L)	1155 ± 4231	74	1011 ± 3633	1477 ± 5218	< 0.01
Sodium (mEq/L)	140 ± 7	41	139 ± 7	140 ± 8	< 0.01
Potassium (mEq/L)	4.2 ± 0.9	45	4.2 ± 0.9	4.1 ± 0.8	0.19
Glucose (mg/dL)	162 ± 84	183	162 ± 94	165 ± 75	0.13
PT-INR	1.2 ± 1.9	582	1.3 ± 2.7	1.1 ± 0.3	0.56
APACHE-II score	13 (8–21)	15	13 (8–21)	13 (9–21)	0.63
Mortality during hospital stay	124 (5.2%)	0	77 (5.1%)	47 (5.4%)	0.77

All categorical variables are shown as n (%). Continuous variables are shown as mean ± standard deviation or median (interquartile range).

*GCS* Glasgow coma scale, *SpO*_*2*_ peripheral oxygen saturation, *BUN* blood urea nitrogen, *AST* aspartate aminotransferase, *ALT* alanine aminotransferase, *PT-INR* prothrombin time-international normalized ratio, *APACHE* acute physiology and chronic health evaluation.

### Assessment of variable importances for the model development

**Figure 2:**
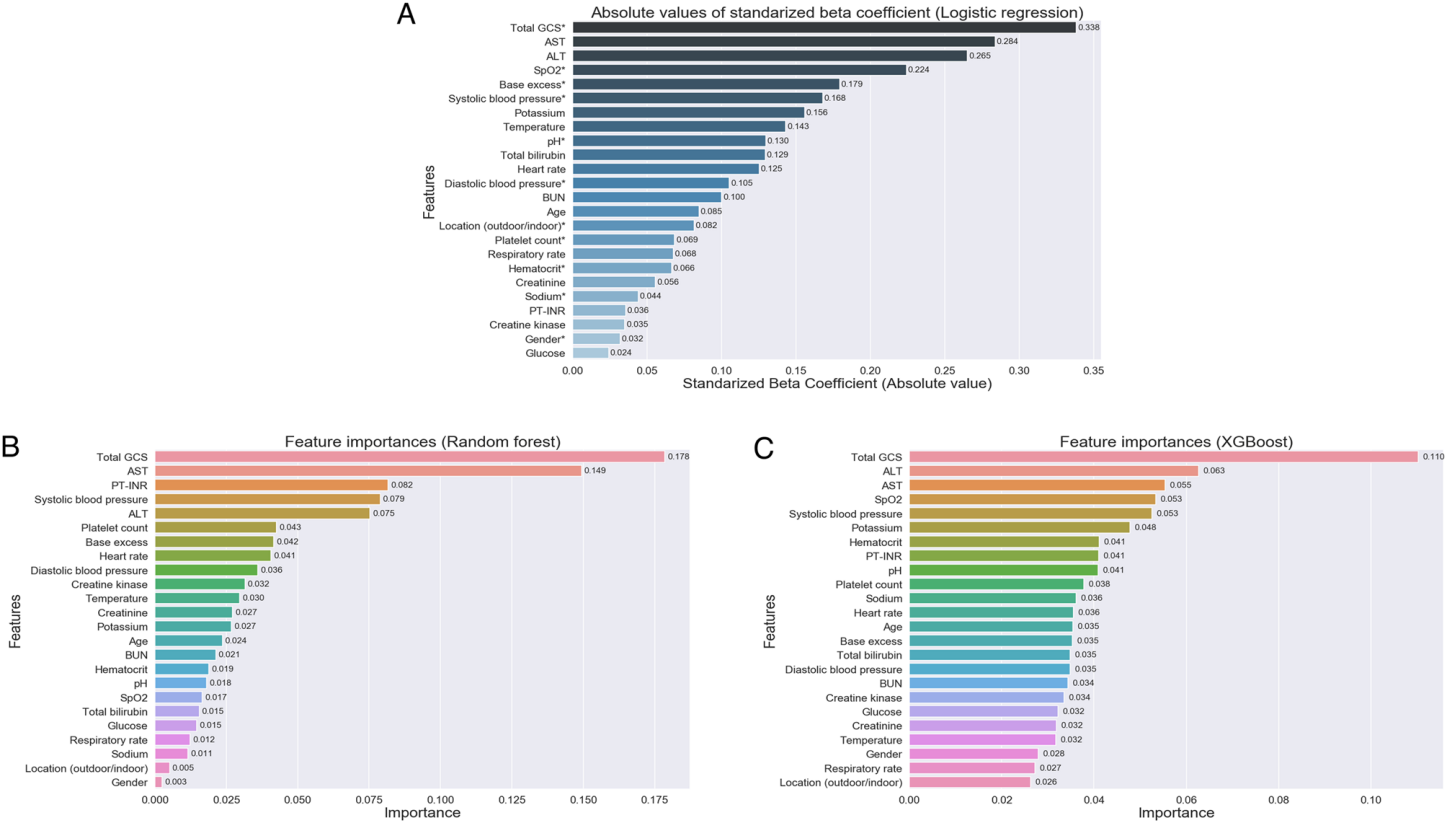
(**A**) Absolute values of standardized beta coefficients for the logistic regression model. (**B**) Feature importances of variables for the random forest model. (**C**) Feature importances of variables for the XGBoost model. Asterisk shows the feature in a positive correlation to the survival outcome. Location (outdoor/indoor)* and gender* refer to outdoor location and male are positive correlation to the survival outcome, respectively. *GCS* Glasgow coma scale, *AST* aspartate aminotransferase, *ALT* alanine aminotransferase, *SpO*_*2*_ oxygen saturation, *BUN* blood urea nitrogen, *PT-INR* prothrombin time/international normalized ratio.

Absolute values of standardized beta coefficients for logistic regression, as well as feature importances for random forest and XGBoost models, were assessed and the results were shown in Fig. 2. In all machine learning models assessed, total GCS score at patients’ hospital arrival was the most essential variable for the prediction of mortality during hospital stay. Both AST and ALT levels in blood were ranked in the top 5 important features in all models. The other key variables to develop the models were SpO_2_ and base excess for the logistic regression, PT-INR and systolic blood pressure for the random forest, and SpO_2_ and systolic blood pressure for the XGBoost.

### Comparison of the accuracy of the models and the reference standard in cross-validation of the training dataset

The training accuracy of machine learning models as the results of cross-validation were 0.852 [SD 0.048] in the logistic regression, 0.841 [SD 0.030] in the support vector machine, 0.918 [SD 0.023] in the random forest, and 0.946 [SD 0.008] in the XGBoost. In contrast, the training accuracy of APACHE-II score was low with 0.773 [SD 0.067].

### Performance analysis of the developed models and the reference standard in the test dataset

**Figure 3:**
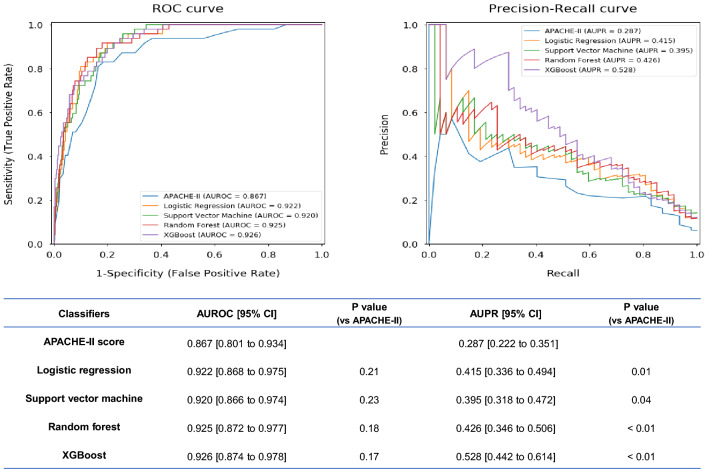
Comparison of ROC curves, PR curves, AUROC, and AUPR among the developed machine-learning models and APACHE-II score for mortality prediction. *ROC* Receiver operating characteristic, *PR* precision-recall, *AUROC* area under the receiver operating characteristic curve, *AUPR* area under the precision-recall curve, *APACHE* acute physiology and chronic health evaluation, *CI* confidence interval.

Figure 3 presents the receiver operating characteristic (ROC) curves and the precision-recall (PR) curves with AUROC and AUPR values of the developed machine learning models and APACHE-II score. Validation of our developed machine learning models showed reliable performance in predicting mortality of heat-related illness, with AUROC values of 0.922 [95% confidence interval (CI) 0.868–0.975] for the logistic regression, 0.920 [CI 0.866–0.974] for the support vector machine, 0.925 [CI 0.872–0.977] for the random forest, and 0.926 [CI 0.874–0.978] for the XGBoost. However, these models could not show statistically significant differences compared to the APACHE-II score with AUROC values of 0.867 [CI 0.801–0.934].

In contrast, there were significantly increased values of AUPR in all developed machine learning models (0.415 [CI 0.336–0.494] for logistic regression, 0.395 [CI 0.318–0.472] for support vector machine, 0.426 [CI 0.346–0.506] for random forest, and 0.528 [CI 0.442–0.614] for XGBoost) compared to APACHE-II score (0.287 [CI 0.222–0.351]).

The confusion matrix and evaluation measures such as sensitivity, specificity, PPV, NPV, and accuracy of the prediction models are shown in Table 2. The logistic regression model demonstrated highest sensitivity of 0.851 [CI 0.749–0.953] and NPV of 0.990 [CI 0.983–0.997] among evaluated classifiers. On the other hand, specificity, PPV and accuracy were highest in XGBoost model with 0.999 [0.996–1.001], 0.875 [0.646–1.104], and 0.953 [0.936–0.965], respectively.

**Table 2:** Comparison of the confusion matrix and evaluation measures among prediction models.

	Predict death	Predict survival	Sensitivity	Specificity	PPV	NPV	Accuracy
**APACH-II score**							
Death	39	8	0.830 [0.722–0.937]	0.778 [0.750–0.807]	0.175 [0.125–0.225]	0.988 [0.979–0.996]	0.781 [0.752–0.808]
Survival	184	646					
**Logistic regression**							
Death	40	7	0.851 [0.749–0.953]	0.848 [0.824–0.873]	0.241 [0.176–0.306]	0.990 [0.983–0.997]	0.848 [0.823–0.872]
Survival	126	704					
**Support vector machine**							
Death	37	10	0.787 [0.670–0.904]	0.846 [0.821–0.870]	0.224 [0.161–0.288]	0.986 [0.977–0.995]	0.843 [0.817–0.866]
Survival	128	702					
**Random forest**							
Death	27	20	0.575 [0.433–0.716]	0.941 [0.925–0.957]	0.355 [0.248–0.463]	0.975 [0.964–0.986]	0.924 [0.902–0.938]
Survival	49	781					
**XGboost**							
Death	7	40	0.149 [0.047–0.251]	0.999 [0.996–1.001]	0.875 [0.646–1.104]	0.954 [0.940–0.968]	0.953 [0.936–0.965]
Survival	1	829					

95% confidence interval were shown in brackets.

*PPV* Positive predictive value, *NPV* negative predictive value.

### Probability calibration curves

Probability calibration curves of prediction models in validation were described in Supplementary Fig. 1. All models were not well-calibrated, indicating that the uncertainty of the predicted probability. XGBoost was underestimated, whereas APACHE-II, logistic regression, support vector machine, and random forest were overestimated for the outcome probabilities.

## Discussion

To our knowledge, the current study is the first to develop and evaluate a machine learning-based prediction model for the prognosis of heat-related illness. In summary, we selected 24 clinical predictors for mortality of heat-related illness from the Japanese heatstroke database. After training these variables using several machine learning algorithms of logistic regression, support vector machine, random forest, and XGBoost, validation of the developed models demonstrated reliable performance with reasonably high AUROC. In comparison of AUPR, all models showed significantly superior performances than APACHE-II as a reference standard.

Heat-related illness can be severe, such as heatstroke, and is induced by an excessively hot and humid environment^2^. Therefore, it is certain that avoiding such an environment would be the best strategy to reduce the poor outcome of this disease. In fact, there has been growing evidence that the environment predisposes people to heat-related illness; in addition, the risk factors for heatstroke have been identified^13,14^. On the other hand, there are few studies on the prognosis of patients who actually develop heatstroke^15,16^. Owing to the lack of a specific mortality prediction tool for heat-related illness, general scoring systems for critically ill patients, such as sequential organ failure assessment (SOFA) and APACHE-II scores, have been commonly used to estimate the severity of this disease^12,17^. The development of specific and reliable prognostic models for heat-related illnesses is anticipated so that clinicians can make an informed decision for optimized treatment. In this context, the current study shows its importance and strength.

Recent evidence has shown the effectiveness of machine learning methods in the development of predictive models in medicine^18,19^. Similarly, we successfully developed a good prognostic model for heat-related illness by using a machine learning algorithm in this study. Referring to the AUROC values, our developed models could not show statistical superiority over the conventional APACHE-II score, even if the models demonstrated higher AUROC values over 0.92 compared to that of APACHE II score with 0.87. However, the current study included only 877 patients for the validation cohort. The limited sample size and lack of statistical power might be the reason why we were not able to find statistical differences in AUROC. More importantly, our data was imbalanced for the outcome with only 5.4% in validation. In the evaluation of performance for imbalanced dataset, AUPR is more appropriate than AUROC because it was specifically fitted for the detection of rare events. Thus, significantly higher AUPR values in the developed models than APACHE-II have encouraged the effectiveness of machine learning to detect rare cases of mortality in heat-related illness. However, calibration plots showed underestimated or overestimated prediction for outcome probability, indicating that these models should be used only for the classification problem.

Our prediction model has the potential to be used in clinical practice. Given that we used only laboratory data and clinical findings at the time of hospital presentation as the predictor variables, the prediction might be used by clinicians as a reference tool for early treatment selection, including internal cooling and cardiopulmonary bypass for severe heat-related illness, which require huge medical costs. Furthermore, the model might be used retrospectively to assess the quality of care for the treatment of heat-related illness. However, we should not use the machine learning model as a definite tool to decide treatment withdrawal.

Notably, body temperature at hospital arrival was not ranked as the highest top five of the mortality predictors selected for machine learning development. In contrast, multiple organ dysfunction indicators were widely chosen, namely, Glasgow coma scale for dysfunction of the central nervous system, systolic blood pressure for circulatory dysfunction, SpO_2_ for respiratory dysfunction, AST and ALT for hepatic failure, PT-INR for coagulopathy, and base excess for metabolic disorders. Inclusion of multiple organ injury markers as parameters is similar to general severity scoring models such as SOFA and APACHE II scores^20,21^; however, variables specifically selected for mortality prediction of heat-related illness might lead to better improvement of predictive performance than the conventional methods. For example, the liver is a common site of tissue injury in heatstroke and causes poor outcome^22,23^. In our machine learning models, AST and ALT levels at hospital arrival were regarded as important predictive values, whereas total bilirubin was included as a hepatic injury indicator in SOFA and no information of hepatic injury in APACHE-II; this difference may affect the predictive ability. In addition, renal dysfunction is relatively common in heatstroke^17,24^. Creatinine level is included in the SOFA and APACHE II scores; however, it was not mainly regarded as the one of important predictors for mortality in our machine learning models, suggesting that complications of renal dysfunction in heat-related illness might not be a strong factor for poor outcome.

Although several variables such as preexisting medical conditions and coagulation abnormalities were recognized as risk factors for the occurrence or poor outcome of heatstroke^25–27^, they were not used in the development of our machine learning models because of the huge amount of missing data in the dataset. The performance of the model might improve if these variables are available for machine learning in the future structured dataset.

Our study has several limitations. First, our prediction model cannot be generalized for application on a global scale. Heat acclimatization can occur in response to heat stress; thus, vulnerability and severity of a heat-related illness can differ depending on the climate in different countries. As we used the Japanese registry database for both training and validation of the model, external validation using databases from foreign countries should be performed in the future. Second, we imputed missing values from the median of each variable. This method is widely used, and is a simple way to impute missing data; however, it could generate bias. Third, the results of evaluation measures for our prediction model demonstrated a wide range of confidence intervals, indicating the uncertainty of the model. This can be attributed to the inadequate total sample size and rare occurrence of outcome (death during hospital stay). However, it is difficult to accumulate data for heat-related illness owing to its seasonal and geographic characteristics. In fact, to our knowledge, there are no larger databases with clinical parameters, including laboratory testing data for heat-related illness, than our heatstroke study registry. Further accumulation of data for such illness is crucial to increase the certainty of the machine learning prediction model. Fourth, we did not focus on the neurologic sequelae of surviving heatstroke patients, which is an important complication of the disease^28^. Although we could not obtain information on the neurological prognosis to be assessed, survival without sequelae should be the primary goal of treatment in real-world practice and thus might exhibit a more significant outcome for the prediction. Fifth, APACHE-II score is not specific to heat-related illness, therefore our study does not guarantee the superiority of machine learning models over simple statistical models which was specifically developed for heat-related illness. Finally, there would be a criticism that machine learning models need a computing device to calculate the results, and a separate model just for the patients with heat-related illnesses would not be realistic. As our selected features were mostly vital signs, laboratory data, and patient background, we suggest the use of machine learning model as a plugin to the electrical hearth record, after the completion of further improvement in the performance and prospective studies for external validation in the future.

## Conclusions

In conclusion, a novel mortality prediction model for patients hospitalized with heat-related illness was developed using a machine learning technique. Although further improvement in the performance quality with increased sample size or inclusion of important variables, as well as prospective validation in a clinical setting are needed, our study demonstrated for the first time the potential of machine learning-based prediction models for heat-related illness.

## Supplementary Information

Supplementary Figure 1.

Supplementary Table 1.
