# Supplementary figures and images for: Machine learning-based mortality prediction model for heat-related illness
Source: Sci Rep. 2021 May 4;11:9501. doi: 10.1038/s41598-021-88581-1 (PMC8096946; doi:10.1038/s41598-021-88581-1)

## Slide 1
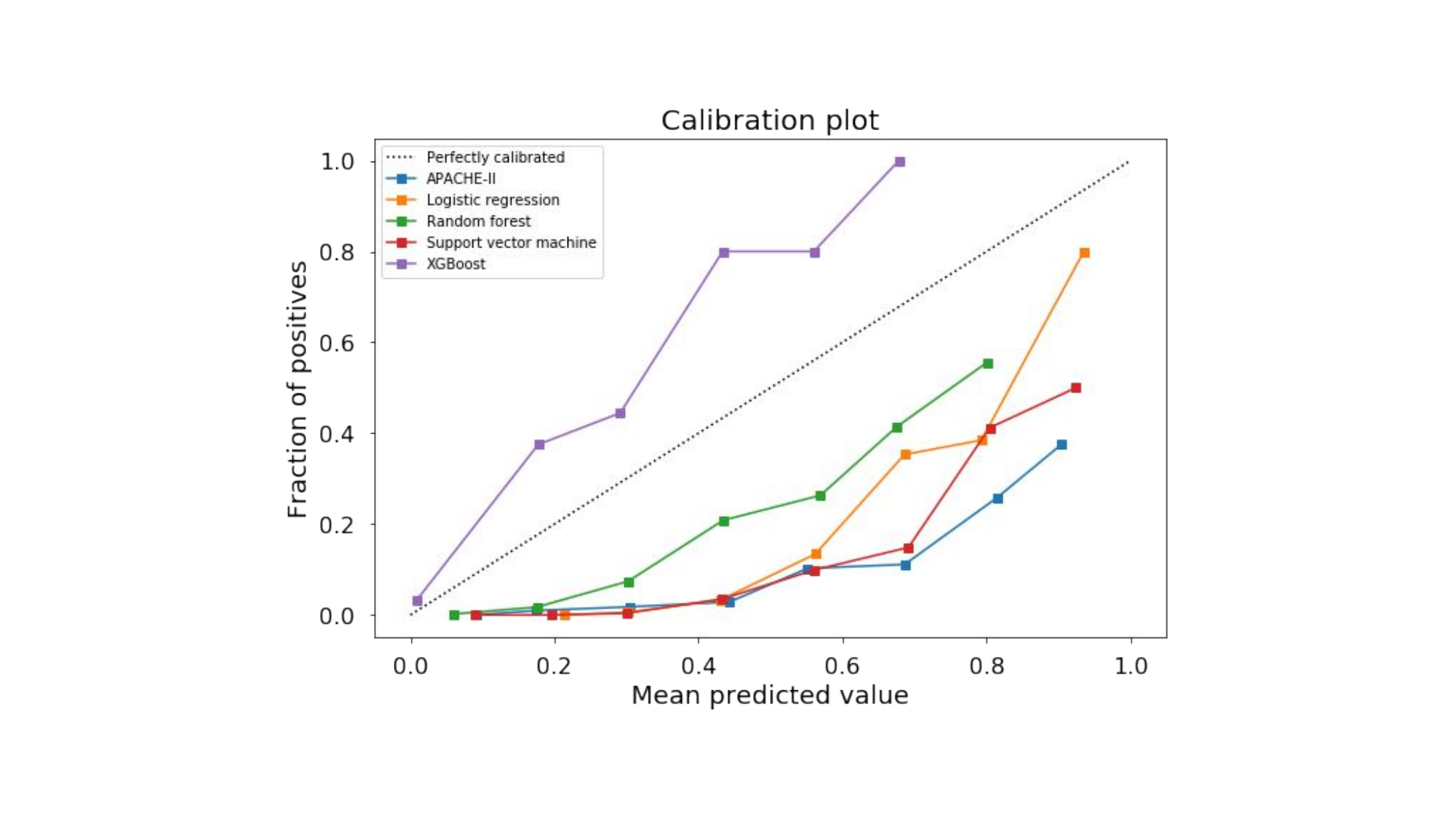

Supplement: Supplementary file 1 — Supplementary Figure 1. [file 41598_2021_88581_MOESM1_ESM.pptx]
