# Supplementary material for: Machine learning-based mortality prediction model for heat-related illness
Source: Sci Rep. 2021 May 4;11:9501. doi: 10.1038/s41598-021-88581-1 (PMC8096946; doi:10.1038/s41598-021-88581-1)
Supplement: Supplementary file 2 — Supplementary Table 1. [file 41598_2021_88581_MOESM2_ESM.docx]

| Models | Hyperparameters |
| --- | --- |
| Logistic regression | C=0.001  Penalty=l2 |
| Support vector machine | Kernel=rbf  C=1.0  Gamma=0.04 |
| Random forest | Estimators=80  Max depth=5 |
| XGBoost | Estimators=100  Max depth=20  Min child weight=0.1  Learning rate=0.1  Subsample=0.5 |

**Supplemental table 1: Key hyperparameters for each machine learning model.**
